# Determinants and Reference Ranges of Serum Immunoglobulins in Middle-Aged and Elderly Individuals: a Population-Based Study

**DOI:** 10.1007/s10875-021-01120-5

**Published:** 2021-09-10

**Authors:** Samer Raza Khan, Layal Chaker, Mohammad Arfan Ikram, Robin Patrick Peeters, Petrus Martinus van Hagen, Virgil Alain Silvester Hovestadt Dalm

**Affiliations:** 1 Department of Internal Medicine, Division of Allergy & Clinical Immunology, Erasmus University Medical Center, Rotterdam, the Netherlands; 2 Department of Epidemiology, Erasmus University Medical Center, Rotterdam, the Netherlands; 3 Department of Internal Medicine, Division of Endocrinology, Erasmus University Medical Center, Rotterdam, the Netherlands; 4 Department of Immunology, Erasmus University Medical Center, Rotterdam, the Netherlands; 5 Department of Internal Medicine, Erasmus University Medical Center, Dr. Molewaterplein 40, 3015 GD Rotterdam, the Netherlands

**Keywords:** Immunoglobulin A, Immunoglobulin G, Immunoglobulin M, Epidemiologic factors, Reference values, Aging

## Abstract

**Purpose:**

In clinical practice, currently one reference range for serum immunoglobulin (Ig) A, G, and M is applied to all adults, although various factors may influence Ig serum levels. Population-based data on determinants of IgA, IgG, and IgM and recommendations for subgroup specific reference ranges are lacking. We aimed to provide an overview of determinants of IgA, IgG, and IgM in community-dwelling middle-aged and elderly individuals and explore determinants that influence Ig reference ranges.

**Methods:**

Within the Rotterdam Study, we performed linear regression analyses for the association of demographic, lifestyle, and cardiovascular factors with serum IgA, IgG, and IgM. We furthermore calculated Ig reference ranges (based on percentiles), both overall and within relevant subgroups.

**Results:**

We included 8768 participants (median age 62 years). IgA and IgG increased non-linearly with higher age (*P* < .0001 for both). Women had lower IgA (beta: − 0.24; 95% confidence interval [95% CI]: − 0.29; − 0.20) and IgG (beta: − 0.33; 95% CI: − 0.44; − 0.23), but higher IgM levels (beta: 0.08; 95% CI: 0.04;0.13) than men. Former and particularly current smoking were associated with lower IgA and IgG (betas between − 0.07 and − 1.03). Higher alcohol consumption was associated with lower IgG (beta for heavy drinking: − 0.70; 95% CI: − 0.91; − 0.48). Corticosteroid use was associated with lower IgG (beta: − 1.12; 95% CI: − 1.58; − 0.66). Associations with cardiovascular factors were heterogeneous and differed between sexes.

**Conclusion:**

Age, sex, smoking, alcohol consumption, corticosteroid use, and cardiovascular factors are determinants that should be considered when interpreting serum Ig levels in middle-aged and elderly individuals and may require adjusted reference ranges.

**Supplementary Information:**

The online version contains supplementary material available at 10.1007/s10875-021-01120-5.

## Introduction

Immunoglobulins (Igs) play an indispensable role in the defense against pathogenic microorganisms as well as in clearing aberrant cells, either directly or through interaction with other components of the immune system. In humans, the five primary classes of Igs are IgA, IgG, IgM, IgD, and IgE (1). Individuals with low levels of serum IgA, IgG, and/or IgM (hypogammaglobulinemia) are prone to infections and various other complications, including autoimmune disorders and malignancies, as can be seen in patients with common variable immunodeficiency, a primary immunodeficiency (PID) characterized by low levels of IgG with low IgA and/or low IgM levels [2–6]. On the other hand, elevated Ig levels have been associated with liver disease, connective tissue diseases, chronic infections, and malignancies among others [7].

Due to the heterogeneous clinical relevance of both low and high serum Ig levels, application of accurately defined reference ranges is essential. In children, age-specific reference ranges of serum Igs are routinely used in clinical practice [8]. For adults, current internationally used reference ranges are based on older studies that recommend one cutoff value for serum Igs, regardless of age or other potential determinants [9]. However, the employed assay, age, and sex are a few of the known factors to influence serum Ig levels [10–16]. Previous studies have shown that Ig levels vary within the adult population and that IgA, IgG, and IgM levels change throughout aging, although results are conflicting on the direction of this association [17–21]. Additionally, other determinants of serum Ig levels in adults, such as lifestyle-related factors, biochemical markers, and drugs, have been identified as well. However, these studies had small sample sizes, only included categorized variables as determinant, did not perform formal regression analyses to test for an association, did not adjust for potential confounders, and/or did not include participants from the general population [22–25]. In a recent systematic review and meta-analysis, we showed that older age and male sex are associated with higher IgA and lower IgM levels. We furthermore reported that smoking and systemic corticosteroid use are associated with lower Ig levels, whereas probiotics, hypertension, alcohol consumption, and acute psychological stress are associated with higher Ig levels [26]. Other cardiovascular risk factors, including anthropometric and serum measurements, have been associated with serum Igs as well [22, 26].

However, no overview exists of potential determinants of serum Ig levels in a general, aging population. We therefore investigated the association of common demographic, lifestyle-related, and cardiovascular risk factors with serum Ig levels in a general population cohort of middle-aged and elderly individuals. Determinants were selected based on biological plausibility, previous literature, and availability in our study cohort. Furthermore, we aimed to define reference ranges of serum IgA, IgG, and IgM in our cohort and assess whether reference ranges differ between subgroups of identified relevant determinants.

## Methods

### Study Design and Participants

This study was embedded in the Rotterdam Study (RS), a large ongoing prospective population-based cohort study including middle-aged and elderly participants from Ommoord, Rotterdam, the Netherlands. Within the RS epidemiological research is performed in various fields, including cardiovascular, endocrine, neuropsychiatric, and respiratory diseases. The RS started in 1990 and had included 14,926 participants by the end of 2008. The RS initially comprised three independent cohorts (RS I, RS II, RS III) that each include re-examination cycles every 3–6 years. In 2016, the fourth cohort started. In addition to the examinations at the research center in Ommoord and home interviews, participants are followed for comorbidities and mortality through continuous automated linkage of the study database with general practitioners’ (GPs’) records. The RS has been approved by the Medical Ethics Committee of the Erasmus Medical Centre (registration number MEC 02.1015) and the Dutch Ministry of Health, Welfare and Sports (Population Screening Act WBO, license number 1071272–159,521-PG). The RS Personal Registration Data collection is filed with the Erasmus Medical Centre Data Protection Officer under registration number EMC1712001. The RS has been entered into the Netherlands National Trial Register and into the World Health Organization (WHO) International Clinical Trials Registry Platform under shared catalogue number NTR6831. Details of the RS have been previously published [27].

For this study, we included all participants from three independent RS cohorts with serum measurements of IgA, IgG, and/or IgM at baseline and written informed consent for follow-up (*n* = 8768).

### Assessment of Serum Immunoglobulins

An immunoturbidimetric assay (Tina-quant® IgA/IgG/IgM Gen. 2, Roche Diagnostics GmbH, Mannheim, Germany) was implemented to measure serum IgA, IgG, and IgM in grams per liter (g/L). All measurements were conducted between 2016 and 2018 in all available baseline samples stored at –80 °C. According to manufacturer’s protocol, the recommended reference ranges for this assay in adults were 0.7–4.0 g/L for IgA, 7.0–16.0 g/L for IgG, and 0.4–2.3 g/L for IgM. These reference ranges are based on the certified reference material for immunochemical measurements of 14 human serum proteins (CRM 470). CRM 470 is an international secondary reference material for adults without provision of age-specific reference ranges [28]. Coefficients of variation (CV) were calculated across all batches (to test assay precision) and between each batch (to identify time trends or batch effects). The CV across batches varied from 1.11 to 5.25% for each Ig. The CV between batches for each Ig varied from 0.61 to 2.85%.

### Baseline Measurements of Potential Determinants and Other Covariates

Smoking status and alcohol consumption were established through questionnaires. Smoking status was defined as current, former, or never smoker. Alcohol consumption was measured in grams per day and was categorized according to WHO guidelines. A daily consumption of 0–10 g/day was considered mild, 10–20 g/day moderate, and > 20 g/day as heavy. Physical activity was reported in metabolic equivalent of task (MET) hours per week and was assessed through validated questionnaires [29, 30]. Anthropometric data was collected during physical examination. Body mass index (BMI) was calculated as weight divided by squared height (kg/m^2^). Waist and hip circumference were measured in centimeters. Blood pressure was measured twice at the right arm in sitting position and the mean value was used for analyses. Hypertension was defined as a blood pressure of ≥ 140/90 mm Hg or as the use of blood pressure lowering medication for the indication of hypertension. Fasting serum samples were collected at the research center in Ommoord. Glucose was measured using the glucose hexokinase method and expressed in millimoles per liter. Triglycerides, HDL, and total cholesterol were measured in millimoles per liter by an automated enzymatic procedure. C-reactive protein (CRP) was measured through an immunoturbidimetric assay in milligrams per liter. Use of oral corticosteroids (ATC code H02), antiepileptic drugs (ATC code N03), and angiotensin-converting enzyme (ACE) inhibitors (ATC code C09) was established during home interviews.

Information on baseline cardiovascular and circulatory diseases (comprising myocardial infarction, revascularization, and stroke) was retrieved through home interviews, linkage with the Dutch Hospital Data (LMR; a national registry of all hospitalizations in the Netherlands), letters of medical specialists, and screening of medical records of GPs. Information was retrieved by trained research assistants and verified by trained independent research physicians according to predefined clinical criteria. Baseline cancer cases were established by two independent physicians based on linkage with the LMR, PALGA (a regional registry of pathology reports), and medical records of GPs. Baseline chronic respiratory disease (chronic obstructive pulmonary disease) was based on pre-bronchodilator spirometry at the research center in Ommoord, or on medical records of respiratory physicians or GPs.

### Statistical Analyses

The association between potential determinants and serum Ig levels was assessed by multiple linear regression models and shown in plots and tables. We standardized continuous determinants for comparison and used two models to examine the association. The first model was unadjusted, and in the second model we adjusted for age, sex, BMI, smoking status, alcohol consumption, and hypertension based on biological plausibility. Predefined stratified analyses were performed by sex. Non-linearity of the association between continuous determinants and Igs was assessed by ordinary least squares linear regression models with restricted cubic splines with 3 knots. Furthermore, residual plots were made for all associations and these did not show heteroscedasticity. Possible outliers were excluded in a sensitivity analysis. The percentage of missing values was ≤ 2.2% for all determinants, with the exception of waist and hip circumference (5.3%), physical activity (14.1%), and alcohol consumption (20.4%). However, complete case analyses showed similar results for these determinants. We applied multivariate imputation by chained equations (5 imputations, 10 iterations) to impute missing values. Reference ranges were defined as the 2.5th and 97.5th percentiles in accordance with widely applied practice in clinical chemistry [31]. Reference ranges of serum Igs were computed for all included participants and for men and women separately. Reference ranges were also computed after exclusion of participants with the top three most burdensome diseases in the elderly, i.e., cardiovascular and circulatory diseases, cancer, and chronic respiratory disease [32]. We furthermore computed reference ranges for 5-year age categories ranging from 45 to ≥ 85 years and for participants < 65 or ≥ 65 years. Reference ranges were also stratified by other determinants if these were significantly associated with serum Ig levels. All analyses were performed in R statistical software versions 3.6.3 and 4.0.0.

## Results

### Study Population Characteristics

We included 8768 participants with a median age of 62.2 years (interquartile range, IQR: 57.4–70.7) and of whom 57% were women. Of these 8768 participants, 8767 had IgA measurements, 8757 had IgG measurements, and 8763 had IgM measurements. Full study population characteristics are shown in Table 1. The median values of IgA, IgG, and IgM were 2.10 g/L, 9.70 g/L, and 0.85 g/L respectively.

**Table 1:** Study population characteristics for 8768 Rotterdam Study participants with immunoglobulin measurements and informed consent for follow-up

Covariate	*N* (%)^a^
IgA, g/L, median (IQR)	2.10 (1.57–2.78)
IgG, g/L, median (IQR)	9.70 (8.30–11.20)
IgM, g/L, median (IQR)	0.85 (0.59–1.23)
Age, years, median (IQR)	62.2 (57.4–70.7)
Sex, female	4995 (57)
Smoking status	
- Current	- 1712 (19.5)
- Former	- 4155 (47.4)
- Never	- 2901 (33.1)
Alcohol consumption^b^	
- None	- 1510 (17.2)
- Mild (0–10 g/day)	- 4673 (53.3)
- Moderate (10–20 g/day)	- 1456 (16.6)
- Heavy (> 20 g/day)	- 1129 (12.9)
Standardized physical activity, MET hours/week, median (IQR)	− 0.16 (− 0.67 to 0.53)
BMI, kg/m^2^, median (IQR)	26.8 (24.5–29.6)
Waist circumference, cm, mean (SD)	93.8 (12.2)
Hip circumference, cm, mean (SD)	104.3 (9.0)
Hypertension	5,345 (61)
Serum glucose, mmol/L, median (IQR)	5.50 (5.10–6.00)
Total serum cholesterol, mmol/L, mean (SD)	5.70 (1.03)
Serum HDL cholesterol, mmol/L, mean (SD)	1.40 (0.41)
Serum triglycerides, mmol/L, median (IQR)	1.33 (1.00–1.82)
Serum CRP, mg/L, median (IQR)	1.50 (0.60–3.40)
Use of systemic corticosteroids	104 (1.2)
Use of antiepileptic drugs	120 (1.4)
Use of ACE inhibitors	1,168 (13.3)

*IgA*, immunoglobulin A; *IgG*, immunoglobulin G; *IgM*, immunoglobulin M; *IQR*, interquartile range; *MET*, metabolic equivalent of task; *BMI*, body mass index; HDL, high-density lipoprotein; *CRP*, C-reactive protein; *ACE*, angiotensin-converting enzyme

^a^Unless stated otherwise

^b^10 g alcohol/day is equivalent to one alcoholic beverage according to World Health Organization guidelines

### Determinants of Immunoglobulins

#### Demographic Factors

**Fig. 1:**
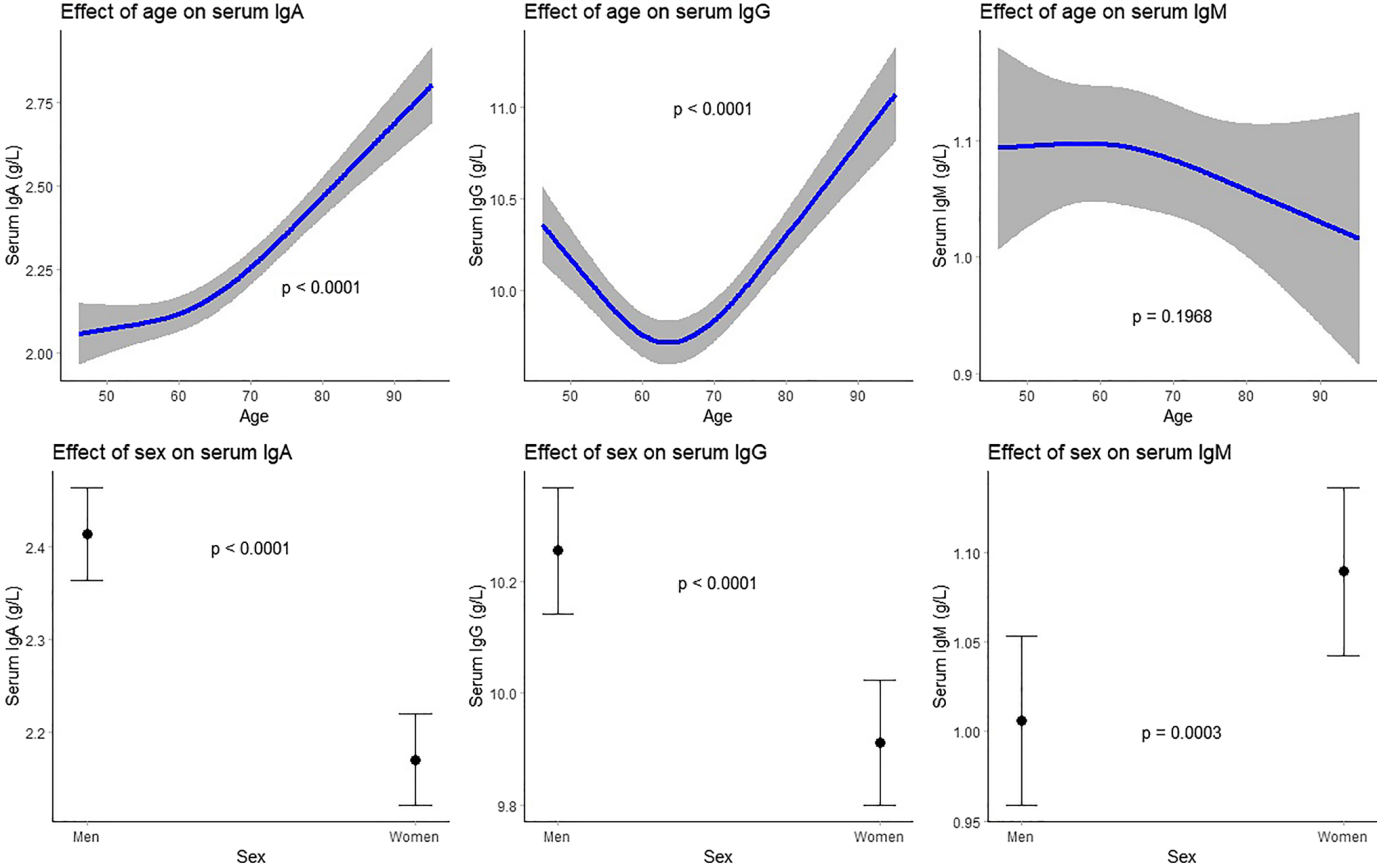
Association between demographic factors and serum immunoglobulin (g/L) levels. Top 3 plots depict association between age and serum IgA, IgG, and IgM respectively with corresponding 95% confidence intervals. The associations have been adjusted for sex, BMI, smoking status, alcohol consumption, and hypertension. Bottom 3 plots depict association between sex and serum IgA, IgG, and IgM respectively with corresponding 95% confidence intervals. The associations have been adjusted for age, BMI, smoking status, alcohol consumption, and hypertension. IgA, immunoglobulin A; IgG, immunoglobulin G; IgM, immunoglobulin M; BMI, body mass index

The associations of age with IgA and IgG (*P* < 0.0001 for both) were non-linear, J- and U-shaped, respectively (Fig. 1). We did not find an association between age and IgM (Table 2). Women had lower IgA (beta: − 0.24; 95% confidence interval [95% CI]: − 0.29; − 0.20) and IgG (beta: − 0.33; 95% CI: − 0.44; − 0.23) levels compared to men, whereas IgM was higher in women (beta: 0.08; 95% CI: 0.04;0.13) (Table 2, Fig. 1).

**Table 2:** Association between standardized covariates and serum immunoglobulin levels

Covariate	IgA (g/L)		IgG (g/L)		IgM (g/L)	
	Beta (95% CI)		Beta (95% CI)		Beta (95% CI)	
	*Model 1*	*Model 2*	*Model 1*	*Model 2*	*Model 1*	*Model 2*
Demographic factors						
Age	Non-linear (*P* < .0001)	Non-linear (*P* < .0001)	Non-linear (*P* < .0001)	Non-linear (*P* < .0001)	− 0.02 (− 0.04;0.01)	− 0.01 (− 0.04;0.01)
Sex						
(female vs male)	− 0.21 (− 0.26; − 0.17)	− 0.24 (− 0.29; − 0.20)	− 0.13 (− 0.23; − 0.03)	− 0.33 (− 0.44; − 0.23)	0.09 (0.04;0.13)	0.08 (0.04;0.13)
Lifestyle factors						
Smoking status						
(former vs never)	− 0.01 (− 0.06;0.04)	− 0.07 (− 0.12; − 0.02)	− 0.27 (− 0.38; − 0.15)	− 0.25 (− 0.37; − 0.13)	− 0.01 (− 0.05;0.04)	0.02 (− 0.03;0.07)
(current vs never)	− 0.31 (− 0.37; − 0.24)	− 0.29 (− 0.35; − 0.22)	− 1.12 (− 1.26; − 0.98)	− 1.03 (− 1.17; − 0.88)	− 0.01 (− 0.07;0.05)	− 0.00 (− 0.06;0.06)
Alcohol consumption						
(mild vs none)	− 0.05 (− 0.13;0.04)	− 0.02 (− 0.10;0.07)	− 0.21 (− 0.36; − 0.06)	− 0.19 (− 0.34; − 0.04)	0.02 (− 0.04;0.08)	0.02 (− 0.05;0.08)
(moderate vs none)	− 0.01 (− 0.11;0.08)	− 0.01 (− 0.10;0.09)	− 0.52 (− 0.71; − 0.32)	− 0.48 (− 0.68; − 0.29)	− 0.01 (− 0.10;0.07)	− 0.00 (− 0.09;0.08)
(heavy vs none)	0.07 (− 0.03;0.16)	0.02 (− 0.08;0.12)	− 0.71 (− 0.91; − 0.50)	− 0.70 (− 0.91; − 0.48)	− 0.07 (− 0.16;0.01)	− 0.05 (− 0.13;0.04)
Physical activity	Non-linear (*P* < .0001)	− 0.04 (− 0.06; − 0.01)	− 0.10 (− 0.16; − 0.04)	− 0.08 (− 0.14; − 0.01)	0.01 (− 0.01;0.04)	0.00 (− 0.02;0.03)
Cardiovascular risk factors						
BMI	0.05 (0.02;0.07)	0.05 (0.03;0.07)	0.09 (0.04;0.14)	0.06 (0.01;0.12)	− 0.02 (− 0.04;0.00)	− 0.02 (− 0.04;0.00)
Waist circumference	0.11 (0.08;0.13)	0.07 (0.02;0.12)	Non-linear (*P* = 0.0180)	− 0.13 (− 0.24; − 0.02)	− 0.04 (− 0.06; − 0.02)	− 0.03 (− 0.07;0.01)
Hip circumference	0.00 (− 0.02;0.02)	− 0.02 (− 0.06;0.02)	0.10 (0.05;0.15)	0.15 (0.06;0.23)	0.00 (− 0.02;0.02)	0.03 (− 0.00;0.07)
Hypertension						
(yes vs no)	0.15 (0.11;0.20)	0.02 (− 0.03;0.07)	0.19 (0.09;0.29)	0.03 (− 0.08;0.14)	− 0.05 (− 0.09; − 0.00)	− 0.03 (− 0.07;0.02)
Glucose	0.10 (0.08;0.12)	Non-linear (*P* < .0001)	− 0.04 (− 0.09;0.01)	Non-linear (*P* = 0.0001)	Non-linear (*P* = 0.0170)	0.00 (− 0.02;0.03)
Cholesterol	− 0.07 (− 0.10; − 0.05)	− 0.06 (− 0.08; − 0.04)	− 0.21 (− 0.26; − 0.16)	− 0.18 (− 0.23; − 0.13)	Non-linear (*P* = 0.0019)	Non-linear (*P* = 0.0006)
HDL cholesterol	− 0.10 (− 0.13; − 0.08)	− 0.07 (− 0.10; − 0.05)	− 0.22 (− 0.27; − 0.17)	Non-linear (P < .0001)	− 0.00 (− 0.02;0.02)	− 0.03 (− 0.05; − 0.01)
Triglycerides	0.02 (0.00;0.05)	Non-linear (*P* = 0.0432)	− 0.05 (− 0.10; − 0.00)	− 0.06 (− 0.12; − 0.01)	Non-linear (*P* = 0.0058)	Non-linear (*P* = 0.0651)
CRP	Non-linear (*P* < .0001)	Non-linear (*P* < .0001)	Non-linear (*P* < .0001)	Non-linear (*P* < .0001)	Non-linear (*P* = 0.0061)	Non-linear (*P* < .0001)
Medication						
Glucocorticoids						
(yes vs no)	− 0.01 (− 0.22;0.20)	-0.12 (-0.32;0.09)	− 0.96 (− 1.43; − 0.49)	− 1.12 (− 1.58; − 0.66)	− 0.03 (− 0.31;0.26)	− 0.01 (− 0.30;0.27)
Anti-epileptics						
(yes vs no)	− 0.19 (− 0.39;0.00)	− 0.20 (− 0.40; − 0.01)	− 0.35 (− 0.78;0.09)	− 0.40 (− 0.83;0.03)	0.10 (− 0.17;0.36)	0.10 (− 0.16;0.37)
ACE inhibitors						
(yes vs no)	0.10 (0.03;0.16)	− 0.00 (− 0.07;0.06)	0.13 (− 0.02;0.28)	− 0.02 (− 0.17;0.14)	− 0.04 (− 0.10;0.02)	− 0.01 (− 0.07;0.06)

Model 1 is unadjusted. Model 2 is adjusted for age, sex, BMI, smoking status, alcohol consumption, and hypertension

*IgA*, immunoglobulin A; *IgG*, immunoglobulin G; *IgM*, immunoglobulin M; *95% CI*, 95% confidence interval; *BMI*, body mass index; *HDL*, high density lipoprotein; *CRP*, C-reactive protein; *ACE*, angiotensin-converting enzyme

#### Lifestyle Factors

**Fig. 2:**
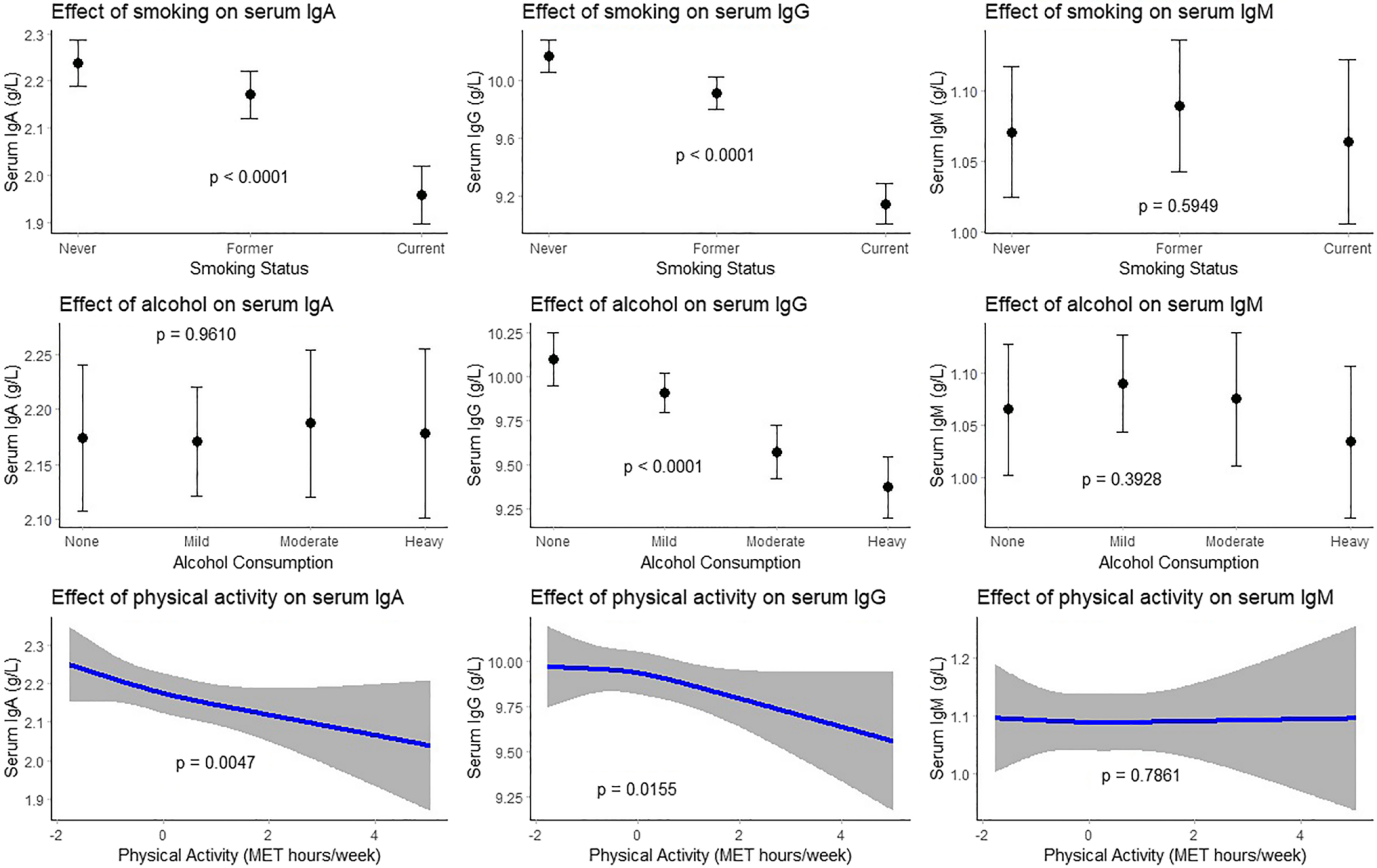
Association between lifestyle factors and serum immunoglobulin (g/L) levels. Top 3 plots depict association between smoking status and serum IgA, IgG, and IgM respectively with corresponding 95% confidence intervals. The associations have been adjusted for age, sex, BMI, alcohol consumption, and hypertension. Middle 3 plots depict association between alcohol consumption and serum IgA, IgG, and IgM respectively with corresponding 95% confidence intervals. The associations have been adjusted for age, sex, BMI, smoking status, and hypertension. Mild consumption was defined as 0–10 g/day, moderate consumption as 10–20 g/day, and heavy consumption as > 20 g/day. According to WHO guidelines, 10 g alcohol/day is equivalent to one alcoholic beverage. Bottom 3 plots depict association between psychical activity and serum IgA, IgG, and IgM respectively with corresponding 95% confidence intervals. The associations have been adjusted for age, sex, BMI, smoking status, alcohol consumption, and hypertension. Psychical activity is depicted as standardized score of metabolic equivalent of task (MET) hours/week. IgA, immunoglobulin A; IgG, immunoglobulin G; IgM, immunoglobulin M; BMI, body mass index; WHO, World Health Organization

Former compared to never smokers had lower serum IgA (beta: − 0.07; 95% CI: − 0.12; − 0.02) and IgG (beta: − 0.25; 95% CI; − 0.37; − 0.13) levels. Current smoking was associated with even lower levels of IgA (beta: − 0.29; 95% CI: − 0.35; − 0.22) and IgG (beta: − 1.03; 95% CI: − 1.17; − 0.88) than never smoking (Table 2, Fig. 2). Alcohol consumption was also associated with lower IgG levels, particularly heavy drinking (beta: − 0.70; 95% CI: − 0.91; − 0.48) (Table 2, Fig. 2). Physical activity was associated with lower serum IgA (beta: − 0.04; 95% CI: − 0.06; − 0.01) and IgG (beta: − 0.08; 95% CI: − 0.14; − 0.01) levels (Table 2, Fig. 2).

#### Cardiovascular Risk Factors

**Fig. 3:**
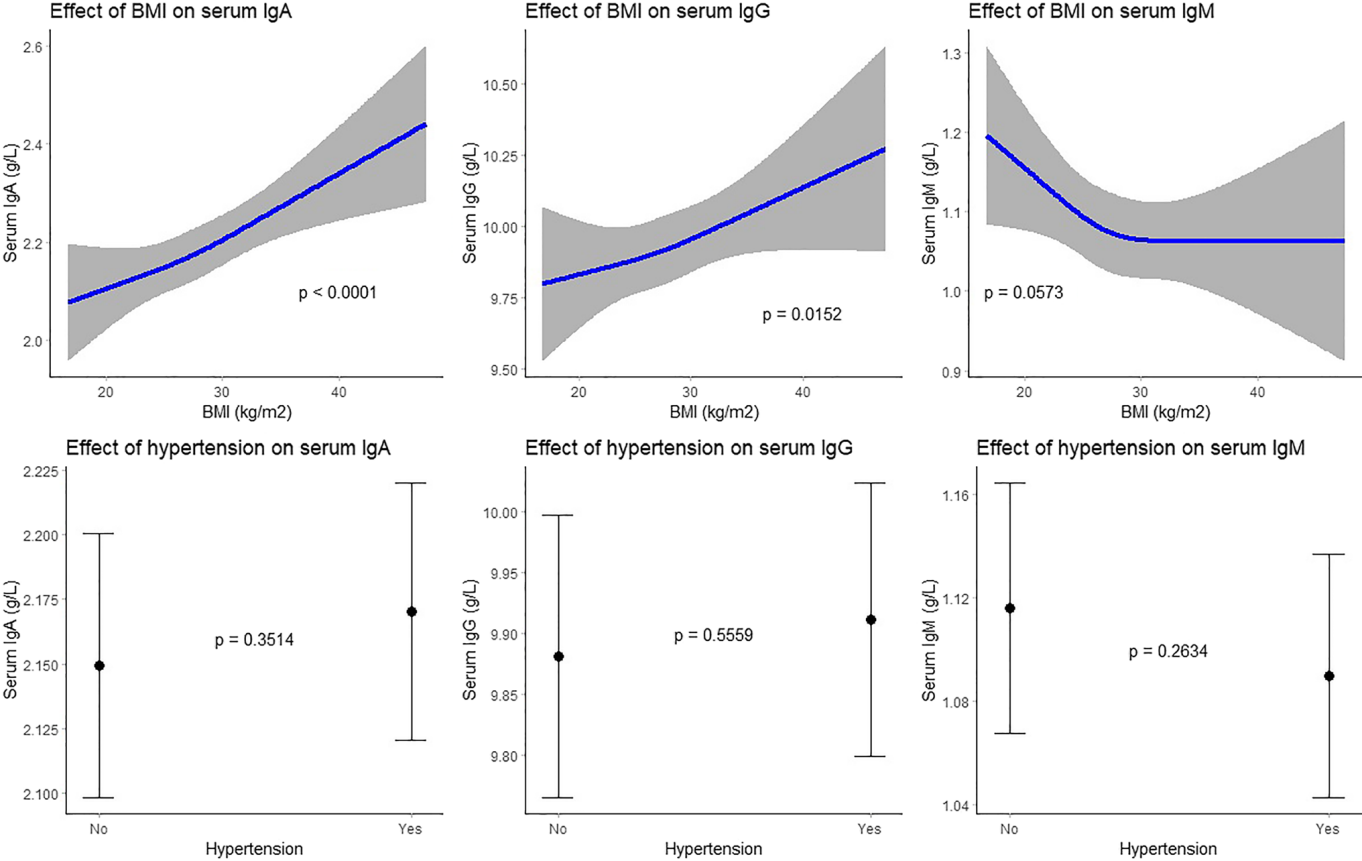
Association between cardiovascular risk factors and serum immunoglobulin (g/L) levels. Top 3 plots depict association between BMI and serum IgA, IgG, and IgM respectively with corresponding 95% confidence intervals. The associations have been adjusted for age, sex, smoking status, alcohol consumption, and hypertension. Bottom 3 plots depict association between hypertension and serum IgA, IgG, and IgM respectively with corresponding 95% confidence intervals. The associations have been adjusted for age, sex, BMI, smoking status, and alcohol consumption. Hypertension was defined as a resting blood pressure of ≥ 140/90 mm Hg or as use of blood pressure lowering agents with the indication of hypertension. IgA, immunoglobulin A; IgG, immunoglobulin G; IgM, immunoglobulin M; BMI, body mass index

Higher BMI was associated with higher serum IgA (beta: 0.05; 95% CI: 0.03;0.07) and IgG (beta: 0.06; 95% CI: 0.01;0.12) levels (Table 2, Fig. 3). Higher waist and hip circumferences were associated with higher IgA (beta: 0.07; 95% CI: 0.02;0.12) and higher IgG (beta: 0.15; 95% CI: 0.06;0.23) levels respectively (Table 2). We found negative associations of total serum cholesterol with IgA (beta: − 0.06; 95% CI: − 0.08; − 0.04) and IgG (beta: − 0.18; 95% CI: − 0.23; − 0.13), of HDL cholesterol with IgA (beta: − 0.07; 95% CI: − 0.10; − 0.05) and IgM (beta: − 0.03; 95% CI: − 0.05; − 0.01), and of serum triglycerides with IgG (beta: − 0.06; 95% CI: − 0.12; − 0.01) (Table 2). Some cardiovascular risk factors were non-linearly associated with serum Igs (Figs. S1-3). Although hypertension was associated with higher IgA and IgG, but lower IgM levels, these associations lost significance in the multivariate model (Table 2, Fig. 3). Higher serum CRP levels were associated with a non-linear increase of IgA, IgG, and IgM (Figs. S1-3).

#### Medication

IgA levels were lower in users of antiepileptic drugs (beta: − 0.20; 95% CI: − 0.40; − 0.01), while IgG was lower in participants that used systemic corticosteroids (beta: − 1.12; 95% CI: − 1.58; − 0.66) (Table 2). No associations were found for ACE-inhibitors with Igs (Table 2).

A graphic overview of identified determinants is presented in Fig. S4. Exclusion of outliers in the residual plots did not change any effect estimates (data not shown).

### Determinants Stratified by Sex

Analyses stratified by sex are shown in Table S1 and Figs. S5 and S6. Most notable differences include the associations with age, smoking, and corticosteroid use.

The association of age with IgA showed a J-shaped increase in women, whereas in men this association was linear (beta: 0.14; 95% CI: 0.10;0.18) (Table S1, Fig. S5). The association of age with IgG was J-shaped in men and U-shaped in women (Table S1, Fig. S6). Women displayed a negative association of age with IgM (beta: − 0.06; 95% CI: − 0.08; − 0.03), while a positive association was found in men (beta: 0.05; 95% CI: 0.01;0.09) (Table S1).

Smoking was associated with lower serum IgG in both sexes, but this was more profound in female current smokers (beta: − 1.17; 95% CI: − 1.36; − 0.97). Use of systemic corticosteroids was associated with lower serum IgG in both sexes and lower serum IgA in men (beta: − 0.35; 95% CI: − 0.71; − 0.00) (Table S1).

## Reference ranges of Immunoglobulins

The overall reference ranges in our population were 0.86–4.76 g/L for IgA, 6.20–15.10 g/L for IgG, and 0.28–2.64 g/L for IgM. In comparison, the assay’s recommended reference ranges were 0.7–4.0 g/L for IgA, 7.0–16.0 g/L for IgG, and 0.4–2.3 g/L for IgM.

Exclusion of the top three most burdensome diseases in the elderly (*n* = 1752) did not change reference ranges (Table 3). Women had slightly lower reference ranges for IgA (0.84–4.58) and IgG (6.10–15.10), but higher for IgM (0.30–2.75) compared to men. No clear trend was observed for age, but overall the upper limit of the reference range of all Igs seemed to increase in the older age groups (Table 3). When stratified by smoking status, IgA (0.81–4.35) and especially IgG (5.80–13.90) were lower in the current smokers (Table 3) and additional analyses showed that this mainly held for female current smokers (Table S2). Alcohol consumption did not influence reference ranges, although heavy drinkers (> 20 g/day) had higher IgA levels (0.98–5.13) (Table 3).

**Table 3:** Reference ranges of serum immunoglobulins (g/L) overall and stratified by determinants

	IgA^a^	IgG^a^	IgM^a^
Overall (*n* = 8768)	0.86–4.76	6.20–15.10	0.28–2.64
After exclusion of top 3 most burdensome diseases of elderly^b^			
(*n* = 7016)	0.86–4.69	6.22–15.00	0.29–2.60
Sex			
- Men (*n* = 3773)	0.91–4.98	6.30–15.10	0.27–2.41
- Women (*n* = 4995)	0.84–4.58	6.10–15.10	0.30–2.75
Age			
- < 65 (*n* = 5285)	0.84–4.47	6.20–14.70	0.31–2.58
- ≥ 65 (*n* = 3483)	0.90–5.30	6.20–15.80	0.26–2.79
Age categories			
- 45–50 (*n* = 488)	0.86–4.24	6.40–14.70	0.32–2.62
- 50–55 (*n* = 898)	0.74–4.49	6.70–15.41	0.33–2.76
- 55–60 (*n* = 1927)	0.84–4.56	6.10–14.30	0.30–2.44
- 60–65 (*n* = 1972)	0.86–4.42	6.12–14.80	0.31–2.66
- 65–70 (*n* = 1134)	0.94–4.85	6.20–14.40	0.28–2.66
- 70–75 (*n* = 947)	0.88–5.11	5.90–15.10	0.27–3.17
- 75–80 (*n* = 742)	0.83–5.25	6.30–16.40	0.25–2.60
- 80–85 (*n* = 412)	0.92–6.27	5.76–17.17	0.21–2.69
- ≥ 85 (*n* = 248)	0.96–6.48	6.75–19.99	0.19–4.57
Smoking status			
- Never (*n* = 2901)	0.86–4.94	6.50–15.90	0.29–2.53
- Former (*n* = 4155)	0.87–4.79	6.30–15.00	0.28–2.68
- Current (*n* = 1712)	0.81–4.35	5.80–13.90	0.27–2.73
Alcohol consumption^c^			
- None (*n* = 1510)	0.84–4.83	6.00–16.03	0.27–2.80
- Mild (0–10 g/day) (*n* = 4673)	0.84–4.69	6.30–15.10	0.29–2.66
- Moderate (10–20 g/day)			
(*n* = 1456)	0.87–4.65	6.14–14.40	0.28–2.57
- Heavy (> 20 g/day) (*n* = 1129)	0.98–5.13	6.10–14.76	0.27–2.46

*IgA*, immunoglobulin A; *IgG*, immunoglobulin G; *IgM*, immunoglobulin M

^a^Reference ranges are 2.5th–97.5th percentiles

^b^Comprises cardiovascular disease (myocardial infarction, revascularization, stroke), cancer, and chronic obstructive pulmonary disease

^c^10 g alcohol/day is equivalent to one alcoholic beverage according to World Health Organization guidelines

**Fig. 4:**
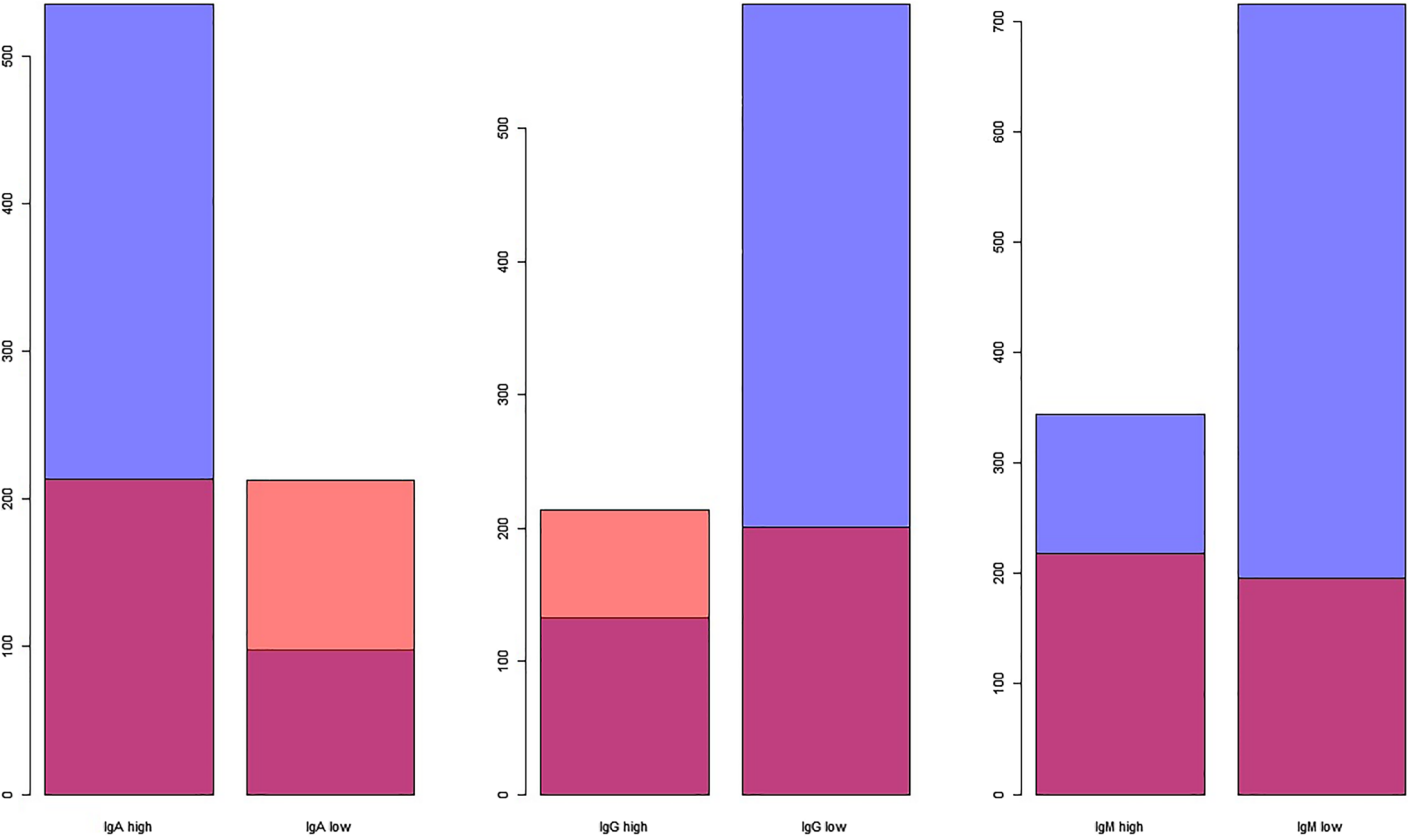
Number of participants with low or high serum immunoglobulin levels according to different reference ranges. Blue depicts the number of participants with serum immunoglobulin levels outside the reference range as recommended by the assay’s manufacturer. Orange depicts the number of participants with serum immunoglobulin levels outside the reference range as computed by us for this population. Red depicts the overlay in participants with low or high immunoglobulin levels according to both reference ranges. IgA, immunoglobulin A; IgG, immunoglobulin G; IgM, immunoglobulin M

For IgA, our reference ranges compared to the assay’s recommended reference ranges yielded fewer participants with hypergammaglobulinemia (214 vs 535), but more with hypogammaglobulinemia (213 vs 98). For IgG however, our reference ranges yielded more participants with hypergammaglobulinemia (214 vs 133) and fewer with hypogammaglobulinemia (201 vs 593), whereas for IgM fewer participants were classified with either hypergammaglobulinemia (218 vs 344) or hypogammaglobulinemia (196 vs 715) (Fig. 4). When taking participants with hypo- and hypergammaglobulinemia together, the largest discrepancy between assay recommended and calculated reference ranges for the entire study population was found for IgM (Table 4). The number of participants with Ig levels in- or outside the reference range for the assay recommended vs our calculated age- and sex-specific reference ranges are depicted in Table S3. For IgA and IgM, the discrepancy was largest in participants ≥ 65 years and in men, whereas for IgG the reference ranges particularly differed in participants < 65 years and in women.

**Table 4:**
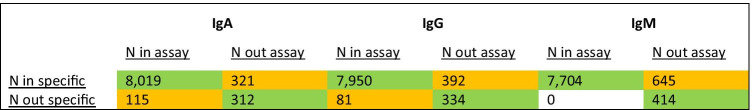
Misclassification of low or high serum immunoglobulin levels according to assay recommended vs Rotterdam Study population specific reference ranges

Numbers depict participants with immunoglobulin levels inside or outside the reference range as recommended by the assay’s manufacturer (0.7–4.0 g/L for IgA, 7.0–16.0 g/L for IgG, and 0.4–2.3 g/L for IgM) or as calculated in our study population (0.86–4.76 g/L for IgA, 6.20–15.10 g/L for IgG, and 0.28–2.64 g/L for IgM)

Green cells display correspondence between reference ranges, while orange cells display discrepancy

*IgA*, immunoglobulin A; *IgG*, immunoglobulin G; *IgM*, immunoglobulin M

## Discussion

We identified age, sex, smoking status, alcohol consumption, physical activity, BMI, serum cholesterol, serum CRP, and systemic corticosteroid use as potential determinants of serum IgA, IgG, and IgM in our general population cohort of middle-aged and elderly individuals. We furthermore established references ranges of serum Igs in our cohort, both overall and stratified by identified relevant determinants. Reference ranges were 0.86–4.76 g/L for IgA, 6.20–15.10 g/L for IgG, and 0.28–2.64 g/L for IgM overall and were furthermore modified by age, sex, smoking status, and alcohol consumption.

IgA showed a J-shaped increase with age, while IgG gradually declined until the age of 60 years and started to increase afterwards. Previous population-based studies showed inconclusive results for the association of age with IgA and IgG, either reporting higher or similar levels at older age. However, these studies were much smaller than the current study, did not adjust for confounders, did not assess non-linearity of the association adequately, or categorized age in their analyses [22, 33–37]. Possible explanations for higher IgA and IgG levels at older age could be a higher ratio of memory to naïve B-cells [38], underlying conditions including rheumatic disorders, infections, or malignancies [7, 39], or monoclonal gammopathy of undetermined significance (MGUS), a highly prevalent disorder of aging [40]. However, higher serum Ig levels at older age could also reflect natural selection, i.e., participants with a better immune function having an increased life expectancy. Proteome analyses showed that healthy centenarians displayed upregulation of B-cell-mediated immune responses, including antibody production, compared to controls from the same geographical region that died prematurely [41].

We found higher IgA and IgG, but lower IgM levels in men compared to women. The results for IgA and IgM are in line with our previous systematic review and meta-analysis and could be due to hormonal differences [26]. In vitro, application of estradiol to human lymphocytes increased B-cell differentiation and number of IgM-producing B-cells, while testosterone had no such effect [42]. Our finding of higher serum IgG levels in men could be explained by the relatively high age of participants in our cohort. Our previous meta-analysis did not show an association between age and IgG, but had included relatively small studies with generally much younger participants. Higher IgG levels in aging men could reflect an underlying inflammatory state. Inflammatory conditions such as cancer and chronic obstructive pulmonary disease are more common in men than women at older age [32].

We also found lower IgA and IgG levels in smokers compared to non-smokers. Nicotine stimulates immunosuppressive hormones including glucocorticoids and catecholamines and can negatively influence B-cell development and Ig production through suppression of the α7-subunit of nicotinic acetylcholine receptors expressed by T- and B-lymphocytes [43, 44]. We furthermore reported lower IgG levels in moderate and heavy alcohol consumers. Two previous population-based studies found similar results [22, 45], although some studies reported unchanged or higher IgG levels in relation to alcohol consumption [46, 47]. However, the latter two studies had small sample sizes (*n* < 60), and only assessed the effect of temporary alcohol admission (either a few hours or weeks) on serum Ig levels. In vitro and in vivo experiments showed that alcohol consumption either stimulates or inhibits lymphocytes depending on the duration and dose of consumption [48].

We confirmed BMI, waist circumference, serum HDL cholesterol, serum triglycerides, and systemic corticosteroid use as determinants [22, 49, 50] and identified total serum cholesterol and serum CRP as new determinants. Metabolic abnormalities such as obesity, a sedentary lifestyle, and elevated serum lipids have been linked to inflammation through various possible pathways including overexpression of inflammatory markers (e.g., tumor necrosis factor α) in adipose tissue and activation of inflammatory kinases by lipids [51]. The immunosuppressive effects of systemic corticosteroids are well recognized and several mechanisms have been described, including reduction in the absolute numbers of circulating T- and B-cells and altered gene transcription in leukocytes, leading to terminal differentiation defects and reduced proliferation of B-cells, impaired B-cell receptor signaling, and reduced expression of all three human Ig loci among others [52, 53]. Higher Ig levels with higher CRP levels could be due to the fact that both reflect an inflammatory state and CRP can be produced by lymphocytes as well [54].

Interestingly, many determinants displayed a similar direction of association with IgA and IgG, suggesting that there may be an effect on immunoglobulin class switch (IgCS). Lipopolysaccharide (LPS) is a well-known inducer of IgCS [55]. Serum LPS is recognized and bound by lipopolysaccharide-binding protein (LBP) [56]. In Caucasians, higher LBP levels were associated with higher BMI, waist-to-hip ratio, and serum glucose and CRP levels, but lower HDL cholesterol levels [57, 58]. Older participants had higher LBP levels as well [58], whereas exercise was associated with a reduction in LBP [59]. Assuming more IgCS in participants with higher LBP levels, these findings are in line with the associations we report for age, cardiovascular risk factors, and physical activity with serum IgA and IgG.

Our age- and sex-specific reference ranges were in line with the reported associations of age and sex with serum Igs. Furthermore, reference ranges of current smokers were lower for IgA and predominantly for IgG than those of former and never smokers. This difference was more apparent in women than in men. Although studies on sex differences in the pharmacodynamics of nicotine are inconclusive, some studies in rats and humans have shown that women metabolize nicotine more slowly and are more sensitive to its effects than men [60].

Our findings urge the application of age- and sex-specific reference ranges of serum Igs in middle-aged and elderly individuals. In our population of healthy elderly, we found lower reference ranges for serum IgG compared to the assay’s recommended reference ranges in adults, yielding fewer participants with hypogammaglobulinemia. This may have important implications for the diagnosis of PIDs at older age, since lower IgG levels represent a physiological rather than pathological state in this population. On the other hand, the threshold for high IgG levels decreases as well, warranting earlier work-up for inflammatory conditions. Current smokers and systemic corticosteroid users had > 1 g/L lower IgG levels compared to non-smokers and non-users of systemic corticosteroids respectively. This is important to take into account for clinicians treating patients with PIDs. It may be advisable to refrain from (long-term) treatment with systemic corticosteroids in a population already struggling with low IgG levels. Alternatively, if such treatment is inevitable, adjustment of intravenous Ig (IVIG) dosage or closer follow-up of serum IgG levels may be suggested. Furthermore, lifestyle changes seem an interesting addition to the treatment of PID patients. Former smokers had a less strong decline in serum IgG than current smokers, suggesting that quitting smoking may have a beneficial influence on serum IgG levels in PID patients as well.

To our knowledge, we are the first to assess the association of various potential determinants and serum Igs in a large population-based cohort of middle-aged and elderly participants. We corrected for a wide number of confounders and displayed non-linear associations of determinants and serum Igs visually. We furthermore provided subgroup-specific reference ranges based on identified determinants. To ensure a healthy study population, we assessed the influence of excluding the top three most burdensome diseases in the elderly. However, our study has some limitations. The RS consists of a mainly Caucasian population; therefore, results might not be generalizable to other populations. Furthermore, we did not have repeated measurements of serum Igs and could not assess potential bidirectional associations with determinants. Moreover, we did not have information on the prevalence of MGUS in our study cohort, which could have an impact on our results, as MGUS is common in the elderly population with an estimated prevalence of 5.3% in individuals aged 70 years and over. Furthermore, translational research is needed to provide more insights into the immunomodulating properties of identified determinants and to provide robust recommendations for clinical practice, including the association between determinants and vaccine or other functional antibody responses. While we assessed the association between determinants and serum immunoglobulins, factors associated with detailed immunophenotyping within the general population could be of future interest as well.

## Conclusion

Age, sex, smoking status, alcohol consumption, corticosteroid use, and cardiovascular factors influence serum immunoglobulin levels in middle-aged and elderly individuals from the general population. Subsequently, our results suggest that currently applied clinical reference values of IgA, IgG, and IgM in adults should be adapted for specific subgroups.

## Supplementary Information

Below is the link to the electronic supplementary material.

Supplementary file1 (DOCX 4597 KB)

## Data Availability

The datasets generated during and/or analyzed during the current study are not publicly available due to participant confidentiality agreements.

## Code Availability

Statistical software codes are available from the corresponding author on reasonable request.
