# Supplementary material for: Determinants and Reference Ranges of Serum Immunoglobulins in Middle-Aged and Elderly Individuals: a Population-Based Study
Source: J Clin Immunol. 2021 Sep 10;41(8):1902–14. doi: 10.1007/s10875-021-01120-5 (PMC8604889; doi:10.1007/s10875-021-01120-5)
Supplement: Supplementary file 1 — Supplementary file1 (DOCX 4597 KB) [file 10875_2021_1120_MOESM1_ESM.docx]

**Supplementary Material**

**Determinants and reference ranges of serum immunoglobulins in middle-aged and elderly individuals: a population-based study**

Samer Raza Khan MD^1,2^, Layal Chaker MD PhD^2,3^, Mohammad Arfan Ikram MD PhD^2^, Robin Patrick Peeters MD PhD^2,3^, Petrus Martinus van Hagen MD PhD^1,4^, Virgil Alain Silvester Hovestadt Dalm MD PhD^1,4^

^1^Department of Internal Medicine, Division of Allergy & Clinical Immunology; ^2^Department of Epidemiology; ^3^Department of Internal Medicine, Division of Endocrinology; ^4^Department of Immunology, Erasmus University Medical Center, Rotterdam, the Netherlands

**Corresponding author**

Virgil A.S.H. Dalm, MD PhD

Department of Internal Medicine, Erasmus MC

Dr. Molewaterplein 40

3015 GD Rotterdam, the Netherlands

Tel: +31 10 70 32690

Fax: +31 10 70 34937

Email: [v.dalm@erasmusmc.nl](mailto:v.dalm@erasmusmc.nl)

| **Table S1. Association between standardized covariates and serum immunoglobulin levels stratified by sex** | | | | | | |
| --- | --- | --- | --- | --- | --- | --- |
| **Covariate** | **IgA (g/L)** | | **IgG (g/L)** | | **IgM (g/L)** | |
|  | Beta (95% CI) | | Beta (95% CI) | | Beta (95% CI) | |
|  | *Men* | *Women* | *Men* | *Women* | *Men* | *Women* |
| **Demographic factors** |  |  |  |  |  |  |
| Age | 0.14 (0.10;0.18) | Non-linear (P <.0001) | Non-linear (P <.0001) | Non-linear (P <.0001) | 0.05 (0.01;0.09) | -0.06 (-0.08;-0.03) |
| **Lifestyle factors** |  |  |  |  |  |  |
| Smoking status  (former vs never)  (current vs never) | -0.04 (-0.13;0.05)  -0.28 (-0.40;-0.17) | -0.09 (-0.16;-0.03)  -0.28 (-0.36;-0.20) | -0.12 (-0.31;0.07)  -0.80 (-1.03;-0.57) | -0.34 (-0.50;-0.18)  -1.17 (-1.36;-0.97) | -0.03 (-0.13;0.08)  -0.02 (-0.15;0.10) | 0.03 (-0.02;0.07)  -0.01 (-0.08;0.05) |
| Alcohol consumption  (mild vs none)  (moderate vs none)  (heavy vs none) | 0.04 (-0.09;0.18)  0.08 (-0.08;0.24)  0.11 (-0.04;0.25) | -0.04 (-0.13;0.06)  -0.06 (-0.17;0.05)  -0.06 (-0.22;0.09) | -0.28 (-0.53;-0.02)  -0.40 (-0.69;-0.11)  -0.61 (-0.90;-0.32) | -0.13 (-0.32;0.05)  -0.58 (-0.85;-0.32)  -0.96 (-1.29;-0.63) | 0.06 (-0.08;0.20)  0.01 (-0.16;0.18)  -0.04 (-0.19;0.12) | -0.00 (-0.07;0.06)  0.01 (-0.09;0.10)  -0.01 (-0.11;0.09) |
| Physical activity | -0.03 (-0.07;0.00) | -0.04 (-0.07;-0.01) | -0.04 (-0.11;0.04) | -0.11 (-0.19;-0.03) | 0.01 (-0.03;0.05) | -0.01 (-0.03;0.02) |
| **Cardiovascular risk factors** |  |  |  |  |  |  |
| BMI | 0.03 (-0.01;0.07) | 0.05 (0.03;0.08) | 0.01 (-0.07;0.10) | 0.09 (0.02;0.15) | -0.04 (-0.09;0.01) | -0.01 (-0.03;0.01) |
| Waist circumference | 0.10 (0.02;0.18) | 0.06 (0.00;0.12) | -0.14 (-0.32;0.04) | -0.11 (-0.24;0.02) | -0.03 (-0.11;0.06) | -0.03 (-0.07;0.01) |
| Hip circumference | 0.02 (-0.05;0.08) | -0.05 (-0.09;0.00) | 0.20 (0.05;0.35) | 0.11 (-0.00;0.23) | 0.00 (-0.07;0.08) | 0.04 (0.01;0.08) |
| Hypertension  (yes vs no) | 0.00 (-0.07;0.08) | 0.04 (-0.02;0.10) | 0.09 (-0.07;0.24) | -0.00 (-0.15;0.15) | -0.06 (-0.14;0.02) | 0.00 (-0.04;0.05) |
| Glucose | Non-linear (P = 0.0009) | Non-linear (P <.0001) | -0.15 (-0.22;-0.08) | Non-linear (P = 0.0798) | 0.01 (-0.03;0.05) | Non-linear (P = 0.1119) |
| Cholesterol | -0.03 (-0.07;0.01) | -0.08 (-0.11;-0.05) | -0.09 (-0.16;-0.01) | Non-linear (P <.0001) | Non-linear (P = 0.0042) | 0.00 (-0.02;0.02) |
| HDL-cholesterol | -0.03 (-0.08;0.02) | Non-linear (P <.0001) | -0.16 (-0.26;-0.07) | Non-linear (P <.0001) | -0.05 (-0.10;0.00) | -0.03 (-0.05;-0.00) |
| Triglycerides | Non-linear (P = 0.0777) | 0.01 (-0.02;0.05) | -0.03 (-0.10;0.03) | -0.09 (-0.17;-0.00) | Non-linear (P = 0.0182) | -0.01 (-0.04;0.01) |
| CRP | Non-linear (P <.0001) | Non-linear (P <.0001) | Non-linear (P <.0001) | Non-linear (P <.0001) | Non-linear (P = 0.0025) | Non-linear (P = 0.0070) |
| **Medication** |  |  |  |  |  |  |
| Glucocorticoids  (yes vs no) | -0.35 (-0.71;-0.00) | 0.01 (-0.24;0.26) | -1.36 (-2.08;-0.65) | -0.97 (-1.58;-0.37) | 0.00 (-0.71;0.72) | -0.04 (-0.23;0.15) |
| Anti-epileptics  (yes vs no) | -0.16 (-0.48;0.17) | -0.24 (-0.47;0.00) | -0.23 (-0.87;0.42) | -0.52 (-1.10;0.05) | 0.10 (-0.51;0.71) | 0.10 (-0.08;0.28) |
| ACE inhibitors  (yes vs no) | -0.03 (-0.13;0.07) | 0.03 (-0.07;0.12) | -0.05 (-0.26;0.16) | 0.01 (-0.21;0.24) | -0.02 (-0.13;0.09) | 0.01 (-0.06;0.08) |
| Betas are adjusted for age, BMI, smoking status, alcohol consumption, and hypertension.  IgA, immunoglobulin A; IgG, immunoglobulin G; IgM, immunoglobulin M; 95% CI, 95% confidence interval; BMI, body mass index; HDL, high density lipoprotein; CRP, C-reactive protein; ACE, angiotensin converting enzyme. | | | | | | |

| **Table S2. Reference ranges^a^ of serum immunoglobulins (g/L) stratified by smoking status and sex** | | | |
| --- | --- | --- | --- |
|  | **IgA** | **IgG** | **IgM** |
| **Men** |  |  |  |
| - Never smoker (n = 791) | 0.86-5.19 | 6.58-15.92 | 0.29-2.12 |
| - Former smoker (n = 2,218) | 0.94-5.01 | 6.40-15.10 | 0.27-2.41 |
| - Current smoker (n = 764) | 0.87-4.55 | 5.91-14.39 | 0.25-2.61 |
| **Women** |  |  |  |
| - Never smoker (n = 2,110) | 0.86-4.81 | 6.50-15.90 | 0.29-2.63 |
| - Former smoker (n = 1,937) | 0.85-4.43 | 6.24-14.80 | 0.31-2.96 |
| - Current smoker (n = 948) | 0.76-4.05 | 5.67-13.53 | 0.30-2.77 |
| ^a^Reference ranges are 2.5^th^ - 97.5^th^ percentiles.  IgA, immunoglobulin A; IgG, immunoglobulin G; IgM, immunoglobulin M. | | | |

| **Table S3. Misclassification of low or high serum immunoglobulin levels according to assay recommended vs age and sex specific reference ranges** | | | | | | |
| --- | --- | --- | --- | --- | --- | --- |
| **Age <65 years** | | | | | | |
|  | **IgA** | | **IgG** | | **IgM** | |
|  | N in assay | N out assay | N in assay | N out assay | N in assay | N out assay |
| N in specific | 4,905 | 120 | 4,810 | 218 | 4,727 | 302 |
| N out specific | 59 | 200 | 74 | 175 | 0 | 253 |
| **Age ≥65 years** | | | | | | |
|  | **IgA** | | **IgG** | | **IgM** | |
|  | N in assay | N out assay | N in assay | N out assay | N in assay | N out assay |
| N in specific | 3,116 | 198 | 3,140 | 174 | 2,977 | 335 |
| N out specific | 54 | 115 | 7 | 159 | 0 | 169 |
| **Male sex** | | | | | | |
|  | **IgA** | | **IgG** | | **IgM** | |
|  | N in assay | N out assay | N in assay | N out assay | N in assay | N out assay |
| N in specific | 3,395 | 194 | 3,447 | 148 | 3,266 | 327 |
| N out specific | 54 | 130 | 34 | 141 | 0 | 178 |
| **Female sex** | | | | | | |
|  | **IgA** | | **IgG** | | **IgM** | |
|  | N in assay | N out assay | N in assay | N out assay | N in assay | N out assay |
| N in specific | 4,623 | 125 | 4,503 | 252 | 4,438 | 313 |
| N out specific | 62 | 184 | 47 | 185 | 0 | 241 |
| Numbers depict participants with immunoglobulin levels inside or outside the reference range as recommended by the assay’s manufacturer (0.7-4.0 g/L for IgA, 7.0-16.0 g/L for IgG, and 0.4-2.3 g/L for IgM) or as calculated in our population based on age and sex. Reference ranges for participants <65 years were 0.84-4.47 g/L for IgA, 6.20-14.70 g/L for IgG, and 0.31-2.58 g/L for IgM. Reference ranges for participants ≥65 years were 0.90-5.30 g/L for IgA, 6.20-15.80 g/L for IgG, and 0.26-2.79 g/L for IgM. Reference ranges for men were 0.91-4.98 g/L for IgA, 6.30-15.10 g/L for IgG, and 0.27-2.41 g/L for IgM. Reference ranges for women were 0.84-4.58 g/L for IgA, 6.10-15.10 g/L for IgG, and 0.30-2.75 g/L for IgM.  Green cells display correspondence between reference ranges, while orange cells display discrepancy.  IgA, immunoglobulin A; IgG, immunoglobulin G; IgM, immunoglobulin M. | | | | | | |


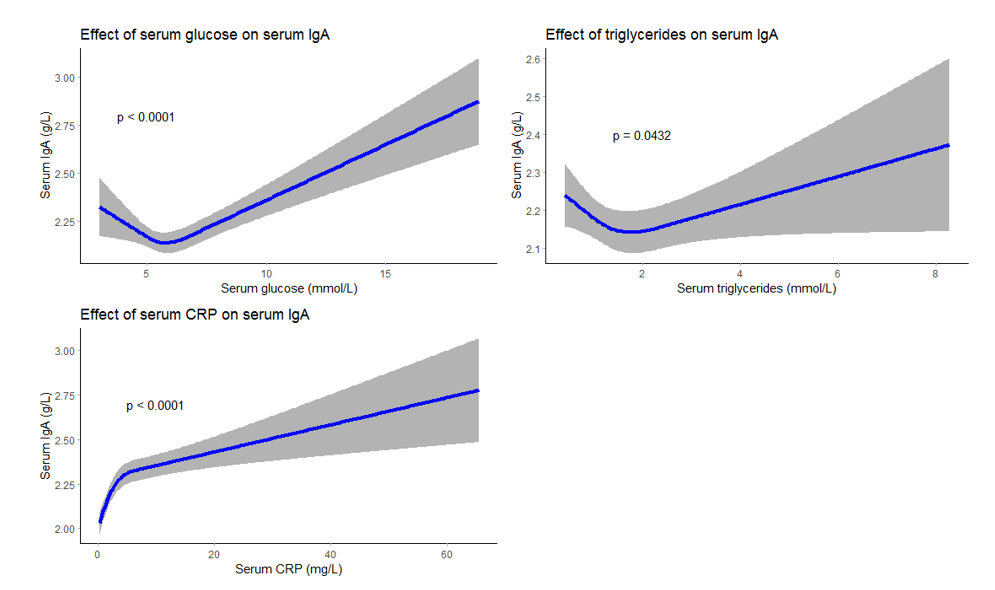


**Fig. S1 Remaining non-linear associations with IgA**

Plots depict non-linear associations of several determinants with serum IgA with corresponding 95% confidence intervals.

Plots are adjusted for age, sex, BMI, smoking status, alcohol consumption, and hypertension.

IgA, immunoglobulin A; CRP, C-reactive protein; BMI, body mass index.


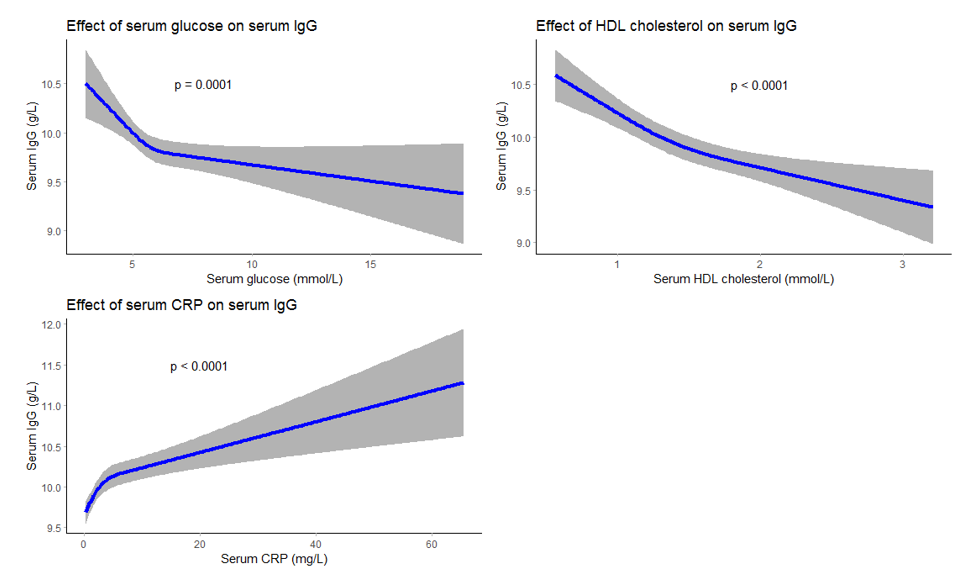


**Fig. S2 Remaining non-linear associations with IgG**

Plots depict non-linear associations of several determinants with serum IgG with corresponding 95% confidence intervals.

Plots are adjusted for age, sex, BMI, smoking status, alcohol consumption, and hypertension.

IgG, immunoglobulin G; HDL, high-density lipoprotein; CRP, C-reactive protein; BMI, body mass index.


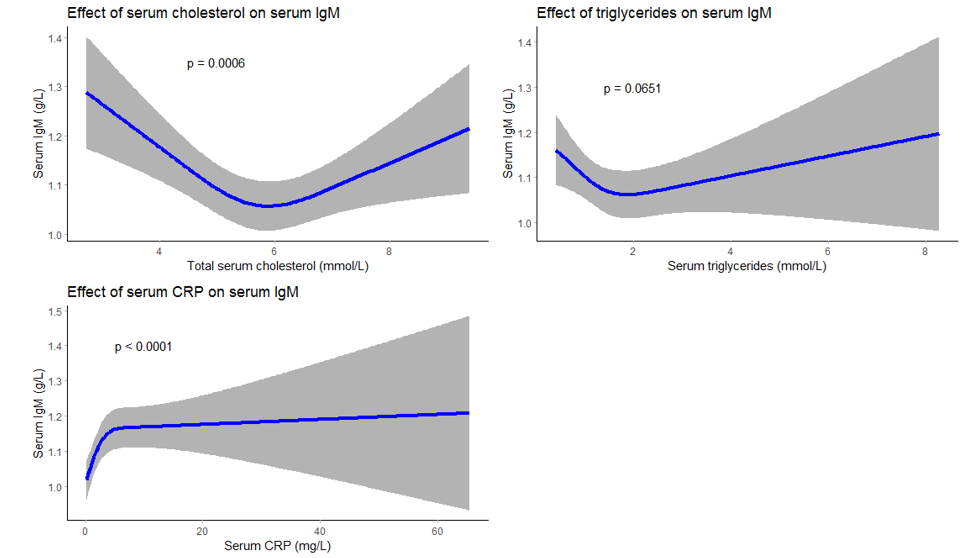


**Fig. S3 Remaining non-linear associations with IgM**

Plots depict non-linear associations of several determinants with serum IgM with corresponding 95% confidence intervals.

Plots are adjusted for age, sex, BMI, smoking status, alcohol consumption, and hypertension.

IgM, immunoglobulin M; CRP, C-reactive protein; BMI, body mass index.


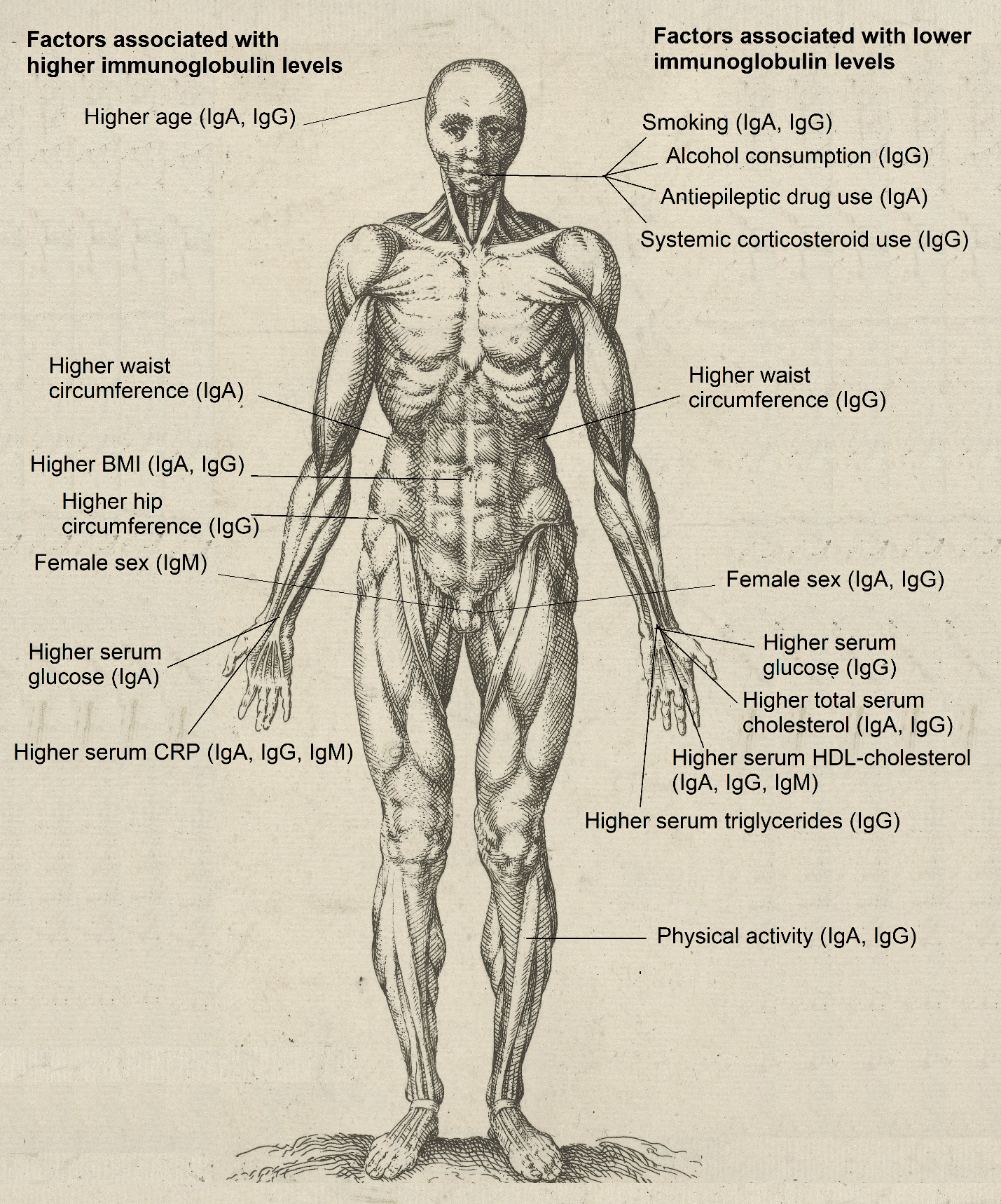


**Fig. S4 Overview of identified determinants**

Figure depicts determinants that were associated with either higher or lower levels of serum immunoglobulin A, G, and/or M.


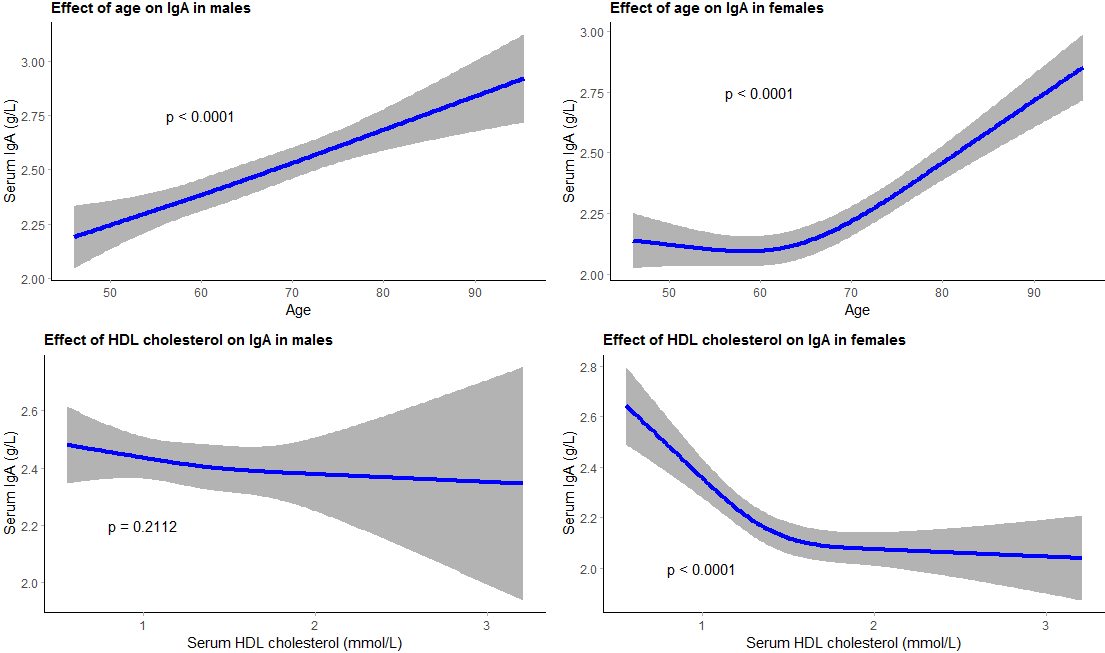


**Fig. S5 Determinants of IgA that differ by sex**

Plots depict the association of several determinants with serum IgA that differed by sex with corresponding 95% confidence intervals. The associations have been adjusted for age, BMI, smoking status, alcohol consumption, and hypertension.

IgA, immunoglobulin A; HDL, high-density lipoprotein; BMI, body mass index.

**
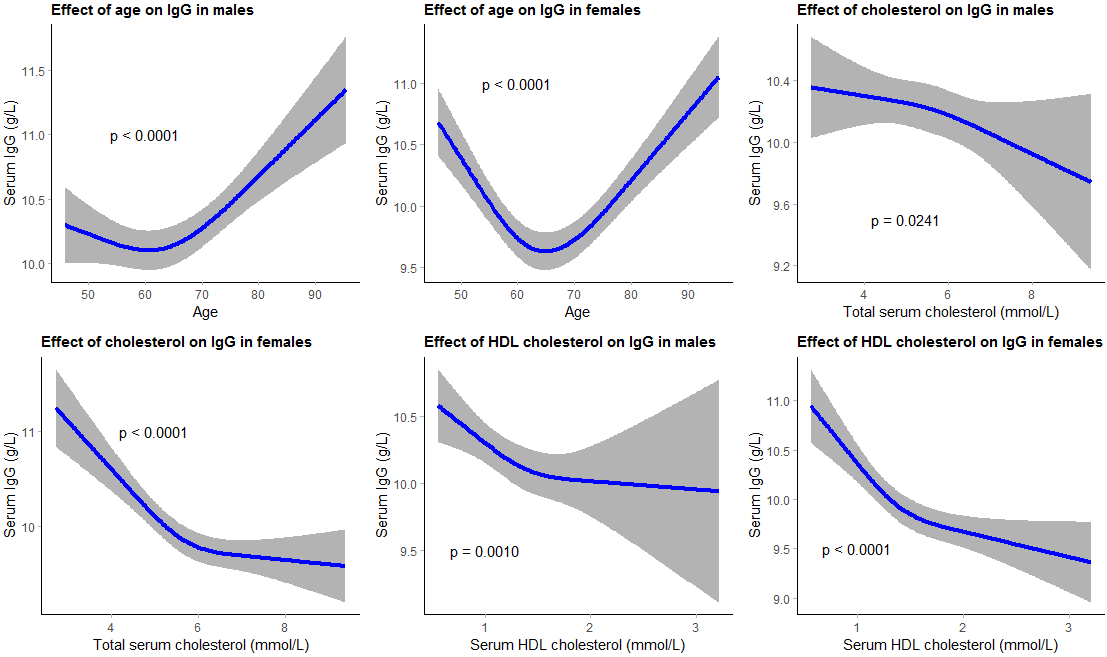
**

**Fig. S6 Determinants of IgG that differ by sex**

Plots depict the association of several determinants with serum IgG that differed by sex with corresponding 95% confidence intervals. The associations have been adjusted for age, BMI, smoking status, alcohol consumption, and hypertension.

IgG, immunoglobulin G; HDL, high-density lipoprotein; BMI, body mass index.
